# Compliance-mediated pressure-independent cerebrovascular regulation: a distinct novel mechanism in youth

**DOI:** 10.1186/s12938-026-01551-4

**Published:** 2026-03-22

**Authors:** Zijie Wang, Youjun Liu, Hao Sun, Tengfei Li, Liyuan Zhang, Guangfei Li, Binxu Yang, Yanjun Gong, Bao Li

**Affiliations:** 1 Department of Biomedical Engineering, College of Chemistry and Life Science, Beijing University of Technology, No. 100 Pingleyuan, Chaoyang District, Beijing, 100124 China; 2 Department of Cardiology, Peking University First Hospital, Beijing, China

**Keywords:** Cerebral blood flow regulation, Breath-holding experiment, Hypercapnia, Breath-holding index (BHI), Cerebrovascular compliance

## Abstract

Cerebrovascular regulatory capacity is essential for maintaining brain stability. Most previous studies have focused on middle-aged and elderly populations, where regulation depends on vasodilation with reduced resistance and elevated blood pressure—the pressure-dependent mechanism. Whether this paradigm applies to younger individuals remains unclear. Given the rising prevalence of cardiovascular and cerebrovascular risks in young adults, elucidating youth-specific mechanisms is of great importance. In this study, standardized breath-holding was used to induce hypercapnia in 52 young volunteers (23.67 ± 1.77 years). Hemodynamic parameters of both middle cerebral arteries (blood pressure, heart rate, and flow velocity) were measured at rest and during hypercapnia. The breath-holding index (BHI), reflecting cerebrovascular regulatory capacity, was calculated, and a hemodynamic model was applied to derive resistance variation coefficient ($R_{v}$), diameter variation coefficient ($D_{v}$), and compliance ($C$), forming a multiparametric framework. In total, 104 unilateral datasets underwent multilevel statistical analysis. Two regulatory patterns were identified: pressure-dependent (*n* = 72) and pressure-independent (*n* = 32). The pressure-independent group showed greater dilation ($D_{v}$: 1.4222 vs. 1.2817, *p* < 0.05) and more stable blood pressure (1.4271 vs. 5.3937 mmHg, *p* < 0.05), achieving comparable cerebrovascular regulatory capacity (${\mathrm{BHI}}$: 1.3327 vs. 1.3907, *p* > 0.05) via enhanced compliance. Strong correlations were observed between rest and task states for blood pressure, heart rate, and flow velocity (all *r* > 0.85, *p* < 0.001). The proposed pressure-independent mechanism challenges the conventional paradigm, highlights individual variability, and offers new insights into cerebrovascular regulatory capacity under hypercapnia.

## Introduction

Cerebrovascular regulatory capacity plays a pivotal role in maintaining brain functional stability. Although the brain accounts for only 2–3% of total body weight, it consumes 15–20% of cardiac output and systemic oxygen consumption [1]. Cerebral autoregulation (CA) stabilizes cerebrovascular function through dynamic adjustments in vascular resistance and flow velocity under a variety of physiological conditions, including fluctuations in blood pressure (BP, 50–170 mmHg), changes in metabolic demand (e.g., neuronal activation), and chemical stimuli (e.g., CO₂ concentration) [2]. The integrity of CA directly influences neurological function and is closely linked to pathological processes such as ischemic stroke and cognitive impairment. Therefore, elucidating the characteristics of cerebrovascular regulatory capacity not only enhances our understanding of fundamental physiological mechanisms but also provides a theoretical basis for early prediction and intervention in related diseases. In this study, BHI and related hemodynamic parameters were used to quantify cerebrovascular regulatory capacity, reflecting the efficiency and characteristics of cerebrovascular regulation.

Hypercapnia is a common physiological stimulus in various pathological conditions (e.g., obstructive sleep apnea syndrome, chronic pulmonary diseases) as well as in breath-holding experiments. By disrupting acid–base balance, hypercapnia induces complex systemic compensatory responses [3–5]. CO₂ diffuses across the endothelium, promotes the release of endothelial-derived relaxing factors, and induces vasodilation, while simultaneously activating chemoreceptors that trigger sympathetic-mediated blood pressure elevation [6–8]. This dual regulatory mode—vasodilation combined with compensatory blood pressure elevation—is widely considered the classical mechanism of cerebrovascular regulation. However, due to individual physiological variability, the applicability of this mechanism may differ across populations. Notably, existing research on cerebrovascular regulation has primarily focused on middle-aged and elderly populations or patients with disease conditions [9–14], whereas systematic characterization in young individuals remains limited. Meanwhile, the prevalence of cardiovascular and cerebrovascular disease risk has reached 15.3% among Chinese individuals aged 20–29 years [15], highlighting a concerning trend of “younger onset”. Against this background, it remains unclear whether the classical vascular regulation paradigm is universally applicable to young populations, or whether alternative or supplementary mechanisms exist.

It is noteworthy that young individuals differ substantially from middle-aged and older populations in terms of vascular structural and functional characteristics. With advancing age, arterial stiffness progressively increases [16, 17], accompanied by a decline in endothelial function [18] and a gradual attenuation of autonomic regulatory capacity [19]. In addition, previous studies have demonstrated that aging is associated with reduced cerebrovascular vasodilatory capacity and decreased efficiency of cerebral blood supply [20]. These multilevel physiological alterations may collectively influence the patterns and efficiency of cerebral blood flow regulation. Therefore, cerebrovascular regulatory models derived from middle-aged, elderly, or diseased populations may not fully reflect the true physiological state of young individuals. In contrast, younger individuals generally exhibit better vascular elasticity and more intact neurovascular coupling capacity, and their regulatory strategies under stress conditions such as hypercapnia may involve different weighting or compensatory patterns. Accordingly, it is necessary to systematically evaluate cerebral blood flow regulatory characteristics in healthy young populations to determine whether distinct mechanisms exist beyond the conventional paradigm.

Furthermore, previous studies have demonstrated that cerebral autoregulation progressively deteriorates with advancing age [21]. Systematic reviews have also indicated that aging leads to an overall decline in autoregulatory function, thereby increasing the dependence of cerebral circulation on changes in perfusion pressure [22]. In a comparative study across the adult lifespan, Xing et al. reported that the coupling between arterial pressure and cerebral hemodynamics becomes stronger with age, suggesting that cerebral blood flow is more readily driven by systemic blood pressure fluctuations [23]. Therefore, in middle-aged and older adults, cerebral blood flow regulation is more likely to exhibit a pressure-dependent regulatory pattern.

Cerebrovascular reactivity (CVR) refers to the capacity of intracranial arterioles and capillaries to maintain cerebrovascular stability through compensatory dilation or constriction in response to physiological or pathological stimuli. CVR is a core index of CBF regulatory capacity [24]. Reduced CVR is typically accompanied by diminished cerebrovascular reserve, significantly increasing the risk of cognitive decline and stroke [25–27]. Despite its clinical and research importance, current CVR assessment methods still face considerable limitations. Magnetic resonance imaging (MRI)-based techniques remain the mainstream, using exogenous CO₂ stimulation combined with blood oxygenation level-dependent (BOLD) or dynamic contrast-enhanced (DCE) approaches [28–30]. Although MRI offers high spatial resolution, it is heavily equipment-dependent, has limited reproducibility (intraclass correlation coefficient ICC = 0.65–0.72), and may be poorly tolerated by some participants [31]. Invasive methods (e.g., transcranial Doppler combined with internal carotid artery catheterization) can alter autonomic activity and shift baseline physiological parameters by more than 15%, further compromising accuracy. By contrast, the breath-holding experiment, owing to its noninvasive nature, operational simplicity, and high ecological validity, has gained increasing attention. This method induces endogenous hypercapnia through voluntary apnea, enabling dynamic acquisition of cerebrovascular regulatory parameters such as the breath-holding index (BHI). It has been validated as an effective approach for assessing cerebrovascular responses to CO₂ elevation [32–35]. Compared with conventional methods, breath-holding does not require exogenous gas delivery or costly equipment, making it particularly suitable for individuals intolerant to invasive procedures. It has thus demonstrated strong potential for clinical screening and research applications [36–40].

Building on this foundation, the present study employed a standardized breath-holding protocol with a dual-modality synchronous recording approach (blood pressure measurement combined with transcranial Doppler ultrasound). The aim was to investigate CBF regulatory mechanisms in young individuals under hypercapnic stress. By constructing a multidimensional quantitative evaluation framework—including hemodynamic theoretical modeling, parameter correlation analyses, inter- and intra-group comparisons, and compliance assessment—we systematically characterized distinct regulatory patterns and their physiological significance. This study not only deepens the understanding of the complexity of cerebrovascular regulatory capacity in young adults, but also provides theoretical support for breath-holding-based noninvasive assessment of cerebrovascular regulatory capacity, with important clinical implications for cerebrovascular health monitoring and personalized intervention.

## Results

Based on bilateral cerebrovascular hemodynamic data from a young cohort (23.7 ± 1.8 years, *n* = 104), this study systematically examined inter-individual differences in cerebrovascular regulatory mechanisms under hypercapnic conditions. ΔBP was defined as the absolute change in MAP between post- and pre-breath-hold measurements, reflecting the intensity of the blood pressure response. The 25th percentile [41–43] of ΔBP fell around 2 mmHg; however, due to clustering of observations at this value and the discrete nature of ΔBP, the actual cumulative proportion slightly exceeded the theoretical percentile. Considering both physiological plausibility and statistical stability, the 30th percentile corresponding to ΔBP = 2 mmHg was adopted as the formal threshold. Accordingly, participants were classified into the pressure-dependent group (ΔBP ≥ 2 mmHg, *n* = 72) and the pressure-independent group (ΔBP < 2 mmHg, *n* = 32). Post-breath-hold ΔBP differed significantly between groups (*p* < 0.05), indicating clear heterogeneity in individual blood pressure responses.

Sensitivity analyses revealed that using the 25th or 35th percentile as grouping thresholds did not materially alter the intergroup differences in blood pressure responses or associated hemodynamic patterns, and the consistency of measurements between resting and task states remained unchanged. These findings support the appropriateness and robustness of adopting the 30th percentile (ΔBP = 2 mmHg) as the classification cutoff in this study.

Statistical power analysis (G*Power 3.1) demonstrated that, with the current sample sizes (pressure-dependent group *n* = 72; pressure-independent group *n* = 32), the effect sizes (Cohen’s d) for key hemodynamic parameters—mean arterial pressure (*MAP*; *MAP*_*t*_ − *MAP*_*r*_, where r denotes resting-state and t denotes task-state), the coefficient of vascular resistance variation (*R*_*v*_), and the coefficient of vascular diameter variation (*D*_*v*_)—were 2.06, 0.92, and 1.00, respectively, indicating large to very large effects. The corresponding statistical power (1 − β) exceeded 0.99 for all comparisons, supporting the statistical robustness of the observed group differences.

### Data quality control and reliability validation

To ensure the reliability of the results, summary statistics of baseline parameters for the total dataset (*n* = 104) and subgroup data are presented in Table 1. Distributional characteristics of hemodynamic parameters under both resting and task conditions were further analyzed (Table 2).

**Table 1:** Analysis of basic information and hemodynamic parameters across different groups

Parameter	Total cohort (*n* = 104)	Pressure-dependent group (*n* = 72)	Pressure-independent group (*n* = 32)
Age	23.67 ± 1.77	23.40 ± 1.55	24.28 ± 2.08
Height (${\mathrm{cm}}$)	167.96 ± 8.14	167.35 ± 8.13	169.33 ± 8.11
Weight (${\mathrm{kg}}$)	60.61 ± 11.28	59.88 ± 11.52	62.23 ± 10.72
${\mathrm{BMI}}$(${\mathrm{kg}} \cdot m^{ - 2}$)	21.35 ± 2.73	21.25 ± 2.86	21.57 ± 2.43
${\mathrm{BHI}}$	1.37 ± 0.44	1.39 ± 0.46	1.33 ± 0.40
$R_{v}$	0.59 ± 0.13	0.62 ± 0.13	0.51 ± 0.10
$D_{v}$	1.33 ± 0.15	1.29 ± 0.14	1.42 ± 0.12
${\mathrm{MAP}}_{t} - {\mathrm{MAP}}_{r}$(${\mathrm{mmHg}}$)	3.81 ± 4.31	5.74 ± 3.64	-0.55 ± 1.74

**Table 2:** Analysis of resting-state and tasking-state parameters across different groups

Group	State	${\mathrm{MAP}}$(${\mathrm{mmHg}}$)	${\mathrm{HR}}$($beats$·$min^{ - 1}$)	$V_{mean}$($cm$·$s^{ - 1}$)
Total cohort (*n* = 104)	Resting state	78.37 ± 8.33	68.96 ± 9.44	62.97 ± 10.87
	Tasking state	82.17 ± 9.24	71.61 ± 10.84	88.72 ± 16.75
Pressure-dependent group (*n* = 72)	Resting state	77.73 ± 8.51	68.65 ± 8.82	63.07 ± 10.90
	Tasking state	83.47 ± 9.66	71.59 ± 10.19	88.88 ± 16.66
Pressure-independent group (*n* = 32)	Resting state	79.81 ± 7.86	69.66 ± 10.84	62.73 ± 10.97
	Tasking state	79.26 ± 7.56	71.65 ± 12.36	88.36 ± 17.21

*HR*, heart rate; *V*_*mean*_, mean flow velocity of the middle cerebral artery

Normality was evaluated using the Kolmogorov–Smirnov test with Lilliefors correction in combination with visual inspection of Q–Q plots for comprehensive assessment. Although minor deviations were indicated for certain variables in statistical testing, visual inspection of Q–Q plots demonstrated that the principal hemodynamic parameters exhibited approximately normal distributions without evidence of systematic skewness. Therefore, given the sample size, the use of parametric statistical methods was considered appropriate and statistically robust.

Bland–Altman analyses showed that the vast majority of data points for key parameters were located within the ±1.96 SD limits of agreement, and no evident systematic bias was observed, indicating good measurement consistency and reliability. In addition, the *BHI*, *R*_*v*_, and *D*_*v*_ values calculated following the standardized breath-hold protocol or derived through fluid dynamic modeling were generally consistent with the trends and numerical ranges reported in previous studies [44, 45], further supporting the reliability of the experimental methodology.

### Hemodynamic differences between pressure-dependent and pressure-independent groups

#### Correlation analysis

**Fig. 1:**
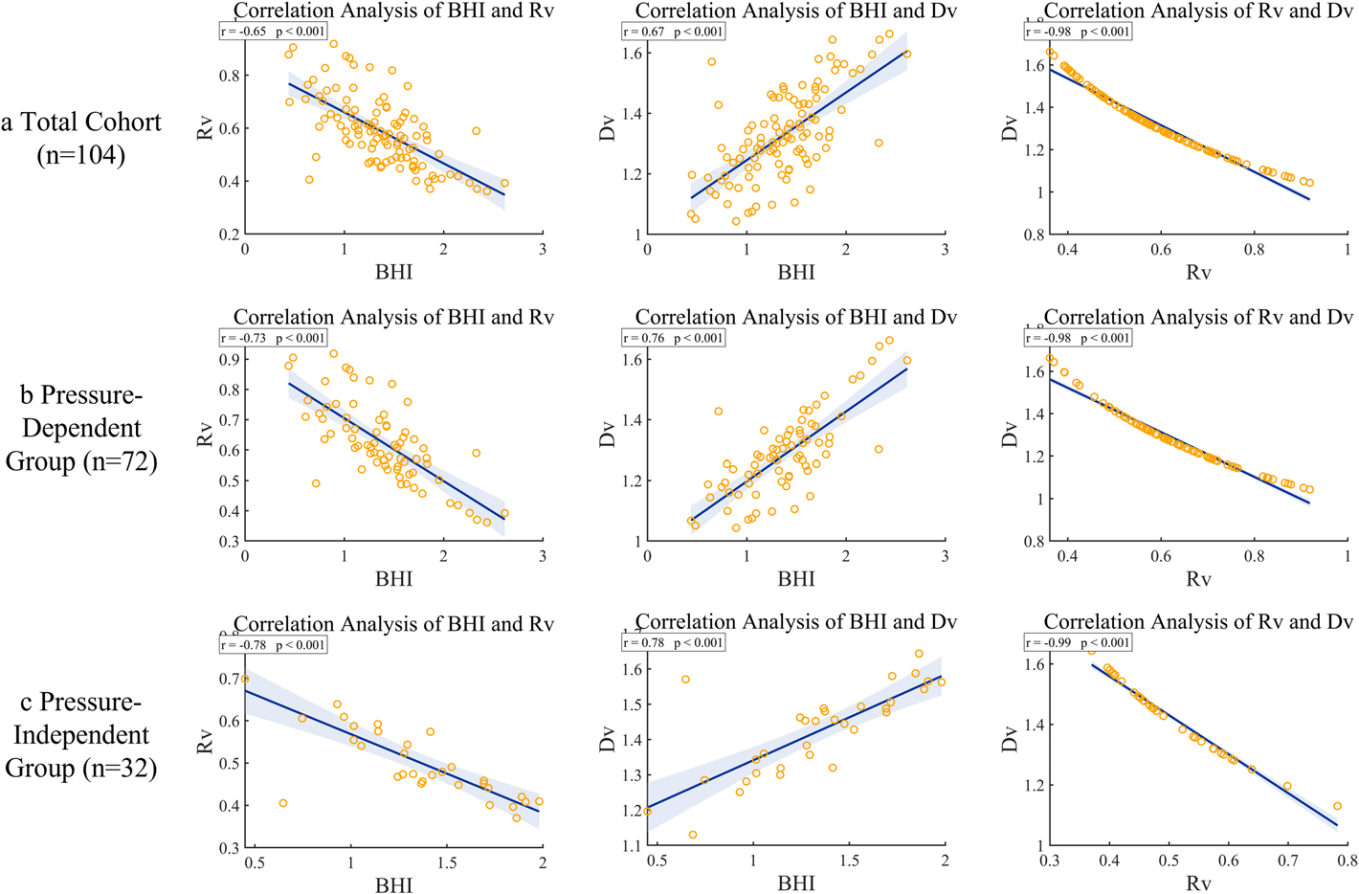
Pearson correlation analysis of ${\boldsymbol{BHI}}$, ${\boldsymbol{R}}_{{\boldsymbol{v}}}$, and ${\boldsymbol{D}}_{{\boldsymbol{v}}}$ across different groups

Pearson correlation analysis was performed to examine systematic relationships among core cerebrovascular hemodynamic parameters. In the total cohort (*n* = 104), the key parameters exhibited highly coordinated changes (Fig. 1a): $BHI$ was significantly negatively correlated with $R_{v}$ (*r* = − 0.6494, *p* < 0.001) and positively correlated with $D_{v}$ (*r* = 0.6688, *p* < 0.001), while $R_{v}$ and $D_{v}$ demonstrated near-perfect inverse covariation (*r* = − 0.9806, *p* < 0.001).

Subgroup analyses revealed that this correlation pattern persisted in both the pressure-dependent and pressure-independent groups. In the pressure-independent group, $BHI$ correlated with $R_{v}$ (*r* = − 0.7821, *p* < 0.001), with $D_{v}$ (*r* = 0.7829, *p* < 0.001), and $R_{v}$ with $D_{v}$ (*r* = − 0.9875, p < 0.001). The pressure-dependent group showed similar trends (*r* = − 0.7339, 0.7627, − 0.9775, all *p* < 0.001).

#### Intergroup differences

**Fig. 2:**
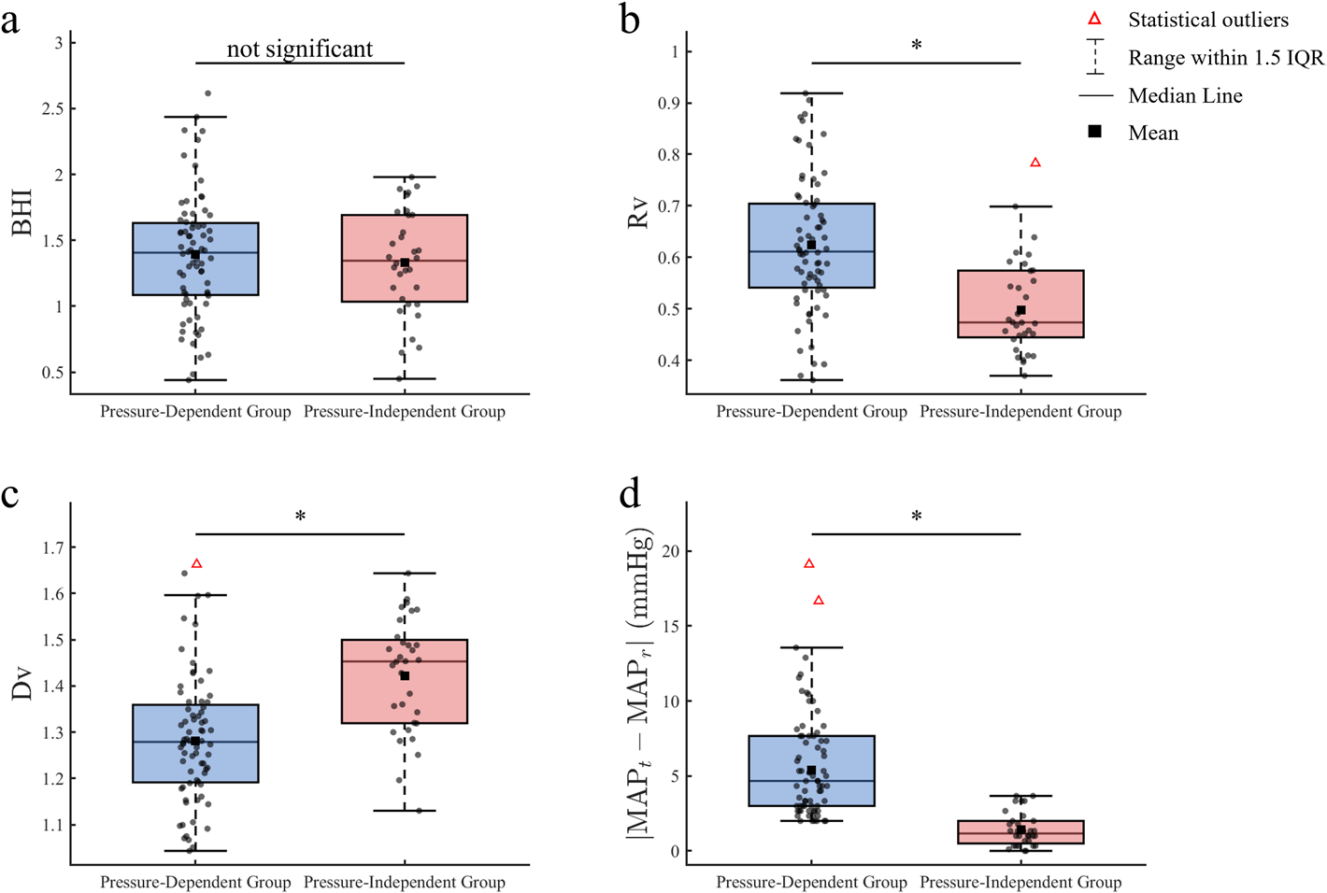
Box plot analysis of key hemodynamic parameters in the pressure-dependent and pressure-independent groups. “*” (*p* < 0.05) indicates statistical significance; “Not significant” (*p* > 0.05) indicates no significant difference

Boxplot comparisons (Fig. 2) demonstrated that $R_{v}$ was significantly lower in the pressure-independent group than in the pressure-dependent group (0.4973 vs. 0.6241, *p* < 0.05), whereas $D_{v}$ was significantly higher (1.4222 vs. 1.2817, *p* < 0.05). $BHI$ showed no statistically significant difference between groups (1.3327 vs. 1.3907, *p* > 0.05).

Post-breath-hold ΔBP differed significantly, with the pressure-independent group exhibiting lower values than the pressure-dependent group (1.4271 mmHg vs. 5.3937 mmHg, *p* < 0.05). No significant differences were observed between groups in rest-state or task-state parameters, nor in baseline demographic characteristics. These findings indicate that basic physiological traits minimally influence individual cerebrovascular regulatory patterns, suggesting that regulatory mechanisms are predominantly driven by dynamic adaptations to hypercapnic stress.

According to the vascular compliance formula (Eq. (8)), the pressure-independent group, characterized by larger vascular diameter changes and smaller BP fluctuations, exhibited higher vascular compliance. This suggests that under hypercapnic stimulation, individuals in this group maintain BP and hemodynamic stability more efficiently through pronounced vasodilation and effective resistance modulation.

### Hemodynamic analysis in rest and task states

#### Parameter correlation analysis

**Fig. 3:**
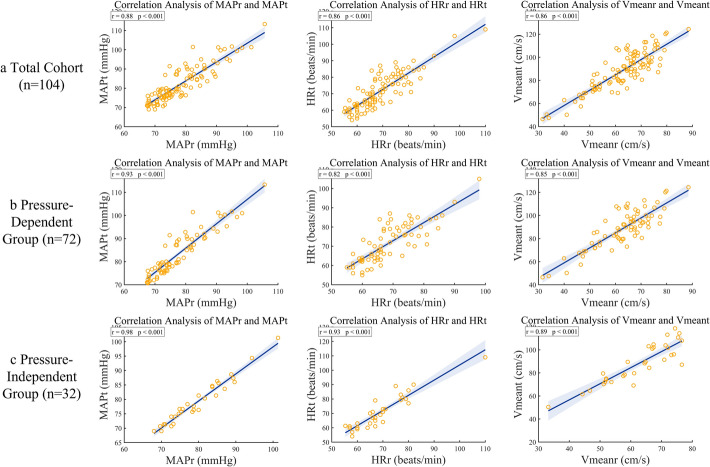
Pearson correlation analysis of hemodynamic parameters between resting and tasking states across different groups

In the total cohort, rest and task measurements of systolic BP (*r* = 0.9262), diastolic BP (*r* = 0.8284), mean arterial pressure (*r* = 0.8850), and heart rate (*r* = 0.8586) were all highly positively correlated (all *p* < 0.001) (Table 3, Fig. 3). Subgroup analyses indicated that both groups maintained significant correlations for *MAP*, *V*_*mean*_, and *HR*. However, correlations were stronger in the pressure-independent group, particularly for mean arterial pressure (*r* = 0.9753 vs. 0.9276, *p* < 0.05) and heart rate (*r* = 0.9256 vs. 0.8165, *p* < 0.05).

**Table 3:** Pearson correlation analysis of hemodynamic parameters between resting and tasking states across different groups

Parameter	Total cohort (*n* = 104)		Pressure-dependent group (*n* = 72)		Pressure-independent group (*n* = 32)	
	*r*	*p*	*r*	*p*	*r*	*p*
$SBP$($mmHg$)	0.9262	< 0.01	0.9380	< 0.01	0.9661	< 0.01
$DBP$($mmHg$)	0.8284	< 0.01	0.8687	< 0.01	0.9495	< 0.01
$MAP$($mmHg$)	0.8850	< 0.01	0.9276	< 0.01	0.9753	< 0.01
$HR$($beats/min$)	0.8586	< 0.01	0.8165	< 0.01	0.9256	< 0.01
$V_{mean}$($cm$/s)	0.8615	< 0.01	0.8485	< 0.01	0.8901	< 0.01

*SBP*, systolic blood pressure; *DBP*, diastolic blood pressure

#### Parameter consistency analysis

**Fig. 4:**
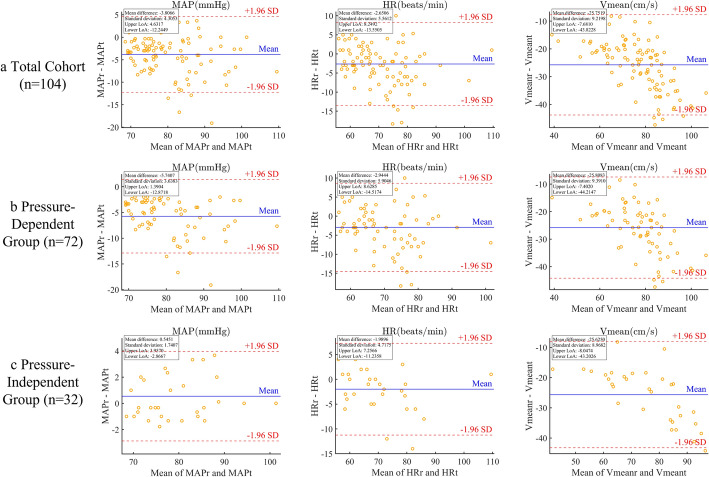
Bland–Altman analysis of hemodynamic parameters between resting and tasking states across different groups

Bland–Altman analyses (Figure 4) showed that measurement errors for *MAP*, *HR*, and *V*_*mean*_ between rest and task conditions conformed to statistical requirements, with approximately 95% of data points falling within ±1.96 SD limits of agreement, and no systematic bias observed. These results validate the consistency and reproducibility of breath-hold measurements across different states.

## Discussion

### Diversity of cerebrovascular regulatory mechanisms and clinical implications

Based on a standardized breath-hold protocol, this study systematically revealed, for the first time in young individuals, two primary cerebrovascular regulatory mechanisms under hypercapnic stress: a pressure-dependent group characterized by BP elevation, and a pressure-independent group characterized by enhanced vascular compliance. The results demonstrated that not all individuals rely on BP elevation to maintain cerebrovascular regulation; some can achieve equivalent regulatory efficiency through increased vasodilation and more efficient resistance modulation, despite minimal BP fluctuations. This finding expands our understanding of cerebrovascular regulatory patterns and provides new evidence for individualized hemodynamic assessment.

For grouping, the 30th percentile (ΔBP = 2 mmHg) was adopted as the classification threshold. Compared with the conventional 25th percentile, this cutoff avoided misclassification due to proximity to device measurement precision and spontaneous physiological fluctuations, while ensuring relatively balanced subgroup sizes, thereby enhancing both statistical power and physiological interpretability.

Correlation analyses further demonstrated commonalities in overall regulatory patterns between the two mechanisms. In the total cohort and both subgroups, $BHI$ was significantly negatively correlated with $R_{v}$, positively correlated with $D_{v}$, and $R_{v}$ was strongly negatively correlated with $D_{v}$, indicating that under CO₂ stimulation, cerebrovascular compensation primarily relies on radius expansion to reduce resistance and maintain homeostasis. *BHI*, as a composite index, effectively quantifies this dynamic regulatory efficiency.

However, significant differences were observed between groups regarding cross-state correlations. The pressure-independent group exhibited markedly higher correlations between rest and task states (*MAP*: *r* = 0.9753 vs. 0.9276; *HR*: *r* = 0.9256 vs. 0.8165; both *p* < 0.05). This high coupling reflects the superior coordination and adaptability of the pressure-independent group in cerebrovascular regulation, indicating a more stable hemodynamic pattern across physiological states and providing a physiological basis for efficient regulation under hypercapnic stress.

Intergroup comparisons revealed individual differences in compensatory pathways. The pressure-independent group showed lower resistance variation ($R_{v}$: 0.4973 vs. 0.6241, *p* < 0.05) and higher diameter variation ($D_{v}$: 1.4222 vs. 1.2817, *p* < 0.05), accompanied by significantly smaller BP fluctuations (1.4271 mmHg vs. 5.3937 mmHg, *p* < 0.05), reflecting higher vascular compliance. In contrast, $BHI$ did not differ significantly between groups (1.3327 vs. 1.3907, *p* > 0.05), indicating that despite differing compensatory pathways, both mechanisms maintain comparable cerebrovascular regulatory capacity.

These findings align with prior studies in older populations, where individuals with lower vascular compliance were more reliant on BP elevation to sustain cerebral blood flow. Our study further demonstrates that a similar pattern exists even in young individuals: those with lower compliance tend to utilize the pressure-dependent mechanism, whereas individuals with higher compliance rely primarily on vasodilation to maintain homeostasis.

Clinically, these findings suggest that individual differences in regulatory patterns should be taken into consideration when assessing cerebrovascular regulatory capacity. For individuals primarily relying on the pressure-dependent mechanism, relatively lower vascular compliance may reflect certain limitations in pressure–volume adaptability. From a theoretical perspective, if such a regulatory characteristic persists over time, it may potentially be associated with hypertension-related cerebrovascular risk factors; however, this inference requires further investigation for confirmation. Mechanistically, this pattern appears to depend more on systemic blood pressure changes rather than intrinsic vasomotor adaptability. Therefore, factors previously reported to improve vascular elasticity, enhance endothelial function, or optimize microcirculatory performance may serve as important directions for future prospective or interventional studies to explore their potential impact on different regulatory patterns.

In contrast, individuals characterized by the pressure-independent mechanism may depend more on vasodilatory capacity and autonomic balance to maintain stable cerebrovascular regulation. From a physiological standpoint, this pattern may reflect relatively favorable vascular adaptability.

In summary, this study demonstrates heterogeneity in cerebrovascular regulatory mechanisms among young individuals and systematically elucidates the physiological basis of two distinct regulatory patterns based on experimental data. As these findings are derived from observational analyses, they provide a novel theoretical perspective for understanding cerebrovascular regulatory strategies, while their implications for individualized health management and disease risk prevention require further evidence-based validation.

### Correlation between rest and task states

This study further validated the predictive value of rest-state parameters for task-state hemodynamic responses. Key indicators, including *MAP*, *HR*, and *V*_*mean*_, exhibited highly significant positive correlations between rest and breath-hold task states (all *p* < 0.001). This suggests that young individuals display highly consistent and predictable cerebrovascular regulation across different physiological states, indicating that rest not only reflects baseline physiological status but also implicitly represents regulatory potential under stress.

Physiologically, this cross-state stability reflects the intrinsic robustness and systemic nature of individual cerebrovascular regulation. First, rest-state characteristics may serve as potential predictors of cerebrovascular response under stress, providing a novel perspective for investigating dynamic regulatory changes. Second, the findings indicate that regulatory mechanisms remain highly consistent across physiological states; in both the total cohort and the two subgroups, rest and task states were significantly correlated, supporting the systemic nature of hemodynamic regulation. This implies that rest-state measurements reflect not only baseline physiology but also latent regulatory capacity under task conditions, illustrating stable cerebrovascular adaptation to external stimuli.

Clinically, the significant correlation between rest and task states offers a foundation for early screening and individualized interventions. Noninvasive rest-state measurements (e.g., TCD or NIRS) may predict cerebrovascular responsiveness under hypercapnic stress, potentially reducing reliance on complex experimental setups and enhancing clinical feasibility and translational value.

Methodologically, Pearson correlation combined with Bland–Altman consistency analysis confirmed the stable correspondence between rest and task states. Key parameters exhibited no systematic bias, with 95% of data points falling within the limits of agreement, supporting the reliability and reproducibility of the experimental results. This methodological validation provides robust statistical support for hemodynamic studies and guidance for future study design.

In conclusion, rest-state measurements can effectively predict task-state hemodynamic changes. These results reveal the systemic and stable nature of cerebrovascular regulation and provide a theoretical basis for developing noninvasive predictive methods and individualized clinical applications.

### Limitations and future work

This study, through a systematic experimental design and data analysis, reveals the diversity of cerebrovascular regulatory mechanisms in young adults and their physiological significance. However, several limitations should be acknowledged and addressed in future research. The primary limitation stems from age and health selection bias. First, the sample consisted of 52 young healthy individuals with a mean age of 23.7 ± 1.8 years, and the limited sample size restricts the generalizability of the findings to middle-aged, older, or clinical populations. Future studies should expand the sample to over 200 participants, covering a broader age range and varying health statuses, to validate the universality of the regulatory patterns. Second, this study mainly relied on hemodynamic indices such as *BHI* and *R*_*v*_, without incorporating measures of sympathetic nervous activity or molecular biomarkers, and did not systematically collect information on physical activity levels or other lifestyle factors, which may potentially influence cerebrovascular regulation. Future research plans to include these quantitative measures to more precisely assess their contributions to different regulatory patterns. In summary, despite limitations in sample size and methodology, this study provides meaningful preliminary insights into cerebrovascular regulatory strategies, and future work will continue to extend these findings through multilevel mechanistic validation and broader population studies.

## Conclusions

Based on a standardized breath-hold protocol, this study systematically revealed that, in addition to the classical BP elevation mechanism, young individuals also exhibit a distinct BP-stable regulatory mechanism centered on vascular compliance, along with its physiological significance under hypercapnic conditions. The results indicate that the traditional pressure-dependent mechanism is not universally applicable to young individuals; a subset of participants can achieve equivalent cerebrovascular regulation while maintaining stable BP through enhanced vasodilation and peripheral resistance modulation, thereby exhibiting higher vascular compliance and more efficient regulatory performance. In contrast, individuals with lower vascular compliance rely more heavily on BP elevation to sustain cerebrovascular homeostasis, resembling regulatory patterns observed in older populations.

Further analyses revealed that rest-state physiological parameters, such as *MAP*, *HR*, and *V*_*mean*_, significantly predict task-state hemodynamic responses, particularly within the pressure-independent group. These findings suggest that rest-state measurements not only reflect baseline physiological status, but also serve as important predictors of cerebrovascular regulatory capacity under stress.

In conclusion, this study not only extends the understanding of cerebrovascular regulation under hypercapnic stress but also, for the first time, systematically identifies a BP-stable regulatory mechanism in young individuals dominated by vascular compliance and possessing predictive value. These findings provide important theoretical support for early cerebrovascular disease screening, individualized assessment, and precision interventions.

## Materials and methods

In this study, standardized 30-s breath-holding tests were used to induce hypercapnia and evaluate the dynamic responses of hemodynamic parameters in the bilateral middle cerebral arteries (MCA). Previous studies have validated that the breath-holding test is significantly correlated with the conventional 5% CO₂ inhalation method in assessing cerebrovascular reactivity (CVR) (Spearman’s ρ = 0.64), confirming its feasibility as an effective approach for cerebrovascular hemodynamic studies [34, 35]. In the present study, a 30-s breath-holding paradigm was adopted as a standardized method for CVR assessment. The duration was not adjusted based on individual breath-holding capacity, thereby eliminating bias from variable stimulus intensities. In addition, unified verbal instructions and pre-test training were provided to minimize technical variations during execution. Although individual lung function may theoretically affect the rate of CO₂ accumulation, such influence on group comparisons is negligible under this standardized protocol.

### Study design

A total of 52 young volunteers were recruited (18 males, 34 females; age 23.67 ± 1.77 years; height 167.96 ± 8.14 cm; weight 60.61 ± 11.28 kg). All participants provided written informed consent after being fully informed about the study and demonstrated adequate compliance and cooperation. The protocol was approved by the Biomedical Research Ethics Committee of Peking University First Hospital.

To minimize experimental bias, participants were instructed to refrain from strenuous exercise, caffeine intake, alcohol, and nicotine exposure for 12 hours prior to testing. The laboratory environment was maintained at 23–24 °C (temperature variation < ±0.5 °C, verified by sensors), with ambient noise and light controlled below 40 dB and 150 lux, respectively. Exclusion criteria included: (1) neurological disorders or impaired brain function; (2) diabetes, pneumonia, bronchial asthma, or other respiratory infections/diseases; (3) hemorrhagic disorders, valvular heart disease, or heart failure; (4) systolic BP ≥ 170 mmHg or diastolic BP ≥ 100 mmHg; (5) frequent premature beats (>10–15 per min), atrial fibrillation, or paroxysmal tachyarrhythmia; (6) any other condition potentially affecting test validity or safety. Brachial blood pressure was measured noninvasively, and bilateral MCA flow velocities were recorded using an ACUSON Antares Ultrasound Imaging System equipped with a PX4-1 probe.

An independent bilateral vessel analysis strategy was adopted, treating left and right MCA as separate statistical units (104 vessel-level datasets in total). This approach, commonly used in vessel-based analyses, increases spatial resolution and enhances statistical robustness [46]. Given that left and right MCAs within the same individual may differ in structure and regulatory responses, separate analysis helps capture subtle regulation patterns.

### Experimental procedure

Prior to testing, demographic data (name, age, sex, height, weight) and medical history (including hypertension, coronary heart disease, valvular disease, congenital heart disease, aortic valve regurgitation, diabetes, pulmonary disorders, thoracic conditions, hemorrhagic disorders, or neurological diseases) were collected. Resting systolic and diastolic BP and heart rate were measured, and informed consent was confirmed.

A two-phase protocol was adopted, consisting of a rest phase and a task phase. Participants were positioned supine throughout.

Rest phase: baseline physiological data were collected, including brachial *MAP*, *HR*, and *V*_*mean*_ using transcranial Doppler (TCD). Each measurement was repeated bilaterally three times, with 1–3 min intervals, and averaged to improve signal-to-noise ratio.

Task phase: at the end of a normal exhalation [36], participants performed a 30-s voluntary breath-hold [40]. Within 4 s after completion, *MAP* and *HR* were recorded, and *V*_*mean*_ was measured again. To avoid residual hypercapnia effects, trials were separated by at least 10 min (≥5 respiratory cycles). Each side was tested three times, and averages were used for analysis.

A 2-MHz Doppler probe (the standard frequency for intracranial vessel TCD, providing adequate penetration) was positioned at the temporal acoustic window and fixed with coupling gel. Probe depth was adjusted to 4–6 cm, optimizing gain and power to achieve stable signals. Pulsed-wave mode was used to record mean flow velocity ($V_{mean}$, cm/s), averaged over 10–20 cardiac cycles to reduce respiratory-cycle interference. Probe position and insonation angle (< 60°) were kept constant, with the sample volume aligned centrally across the vessel lumen [47]. Participants were instructed to remain relaxed and avoid unnecessary movements to ensure measurement reliability.

### Multidimensional hemodynamic parameter assessment

**Fig. 5:**
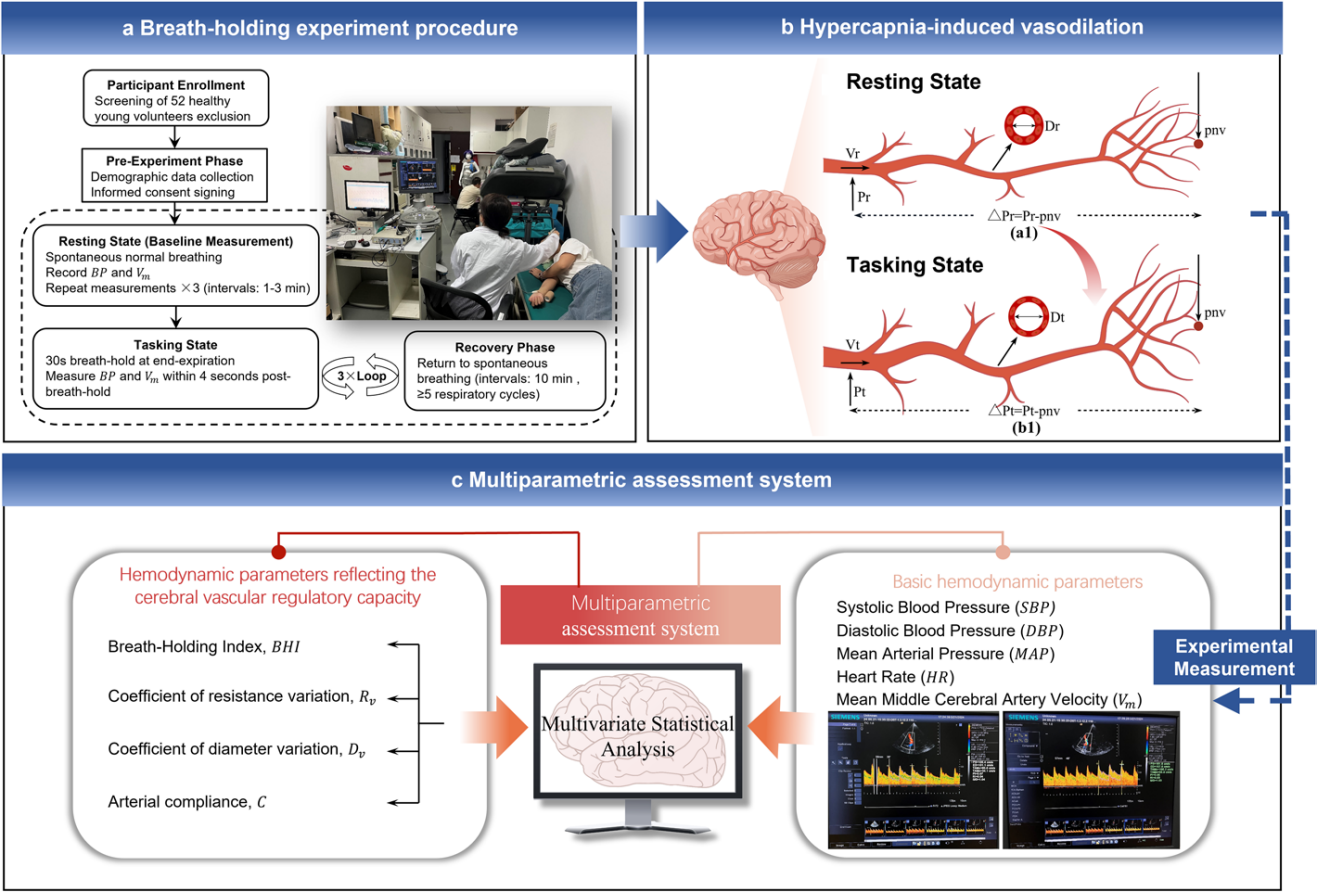
Assessment of cerebral blood flow regulation. **a** breath-holding experiment procedure. **b** Hypercapnia-induced vasodilation. Here, ${\boldsymbol{P}}$ denotes the mean arterial pressure, ${\boldsymbol{V}}$ represents the blood flow velocity, $\Delta P$ indicates the cerebral arterial pressure drop, ${\boldsymbol{pnv}}$ refers to the mean venous pressure (normalized to 20 mmHg), ${\boldsymbol{D}}$ is the diameter of cerebral arteries, and the subscripts ${\boldsymbol{r}}$ and ${\boldsymbol{t}}$ correspond to the resting and task states, respectively. **c** Multiparametric assessment system

Based on the breath-holding test (Fig. 5a), hemodynamic parameters were derived to evaluate cerebrovascular regulation. At rest (Fig. 5b-a1), MCA maintained a steady state, whereas task-induced CO₂ elevation during breath-holding triggered vasodilation (Fig. 5b-b1). Fundamental parameters included brachial MAP and MCA mean flow velocity ($V_{mean}$). These were integrated with hemodynamic indices derived from fluid mechanics to quantify regulation, including the breath-holding index ($BHI$), resistance variation coefficient ($R_{v}$), diameter variation coefficient ($D_{v}$), and vascular compliance ($C$), as illustrated in Fig. 5c.

The calculations were based on the following models:

Breath-holding index (BHI): quantifies cerebrovascular response to hypercapnia [34]:

1 $$ \begin{array}{*{20}c} {{\mathrm{BHI}} = \left( {\left( {V_{{{\mathrm{mean}}\_{\mathrm{30sec}}}} - V_{{{\mathrm{mean}}\_{\mathrm{basal}}}} } \right)/V_{{{\mathrm{mean}}\_{\mathrm{basa}}l}} } \right)/\left( {30/100} \right)} \\ \end{array} , $$

where $V_{{{\mathrm{mean}}\_30sec}}$ is MCA velocity after 30 s of breath-holding (cm/s), and $V_{{{\mathrm{mean}}\_basal}}$ is baseline velocity.

Resistance variation coefficient ($R_{v}$): reflects dynamic adjustments in vascular resistance:

2 $$ \begin{array}{*{20}c} {R_{v} = \frac{{R_{t} }}{{R_{r} }} = \frac{{\Delta {\mathrm{MAP}}_{t} *Q_{r} }}{{\Delta {\mathrm{MAP}}_{r} *Q_{t} }} = \frac{{\Delta {\mathrm{MAP}}_{t} *V_{{{\mathrm{meanr}}}} *A_{r} }}{{\Delta {\mathrm{MAP}}_{r} *V_{{{\mathrm{meant}}}} *A_{t} }} = \frac{{\Delta {\mathrm{MAP}}_{t} *V_{{{\mathrm{meanr}}}} *\left( {D/2} \right)_{r}^{2} }}{{\Delta {\mathrm{MAP}}_{r} *V_{{{\mathrm{meant}}}} *\left( {D/2} \right)_{t}^{2} }} = \frac{{\Delta {\mathrm{MAP}}_{t} *V_{{{\mathrm{meanr}}}} }}{{\Delta {\mathrm{MAP}}_{r} *V_{{{\mathrm{meant}}}} *\left( {D/2} \right)_{v}^{2} }}} \\ \end{array} , $$

where $\Delta P = R \cdot Q$ [48], $R$ is vascular resistance, $Q$ is arterial flow, and $A$ is vessel cross-sectional area.

Pressure difference equations [49]:

3 $$ \begin{array}{*{20}c} {\Delta {\mathrm{MAP}}_{t} = {\mathrm{MAP}}_{t} - {\mathrm{pnv}}} \\ \end{array} , $$

4 $$ \begin{array}{*{20}c} {\Delta {\mathrm{MAP}}_{r} = {\mathrm{MAP}}_{r} - {\mathrm{pnv}}} \\ \end{array} . $$

Assuming Newtonian fluid and laminar flow, resistance satisfies Poiseuille’s law [48]:

5 $$ \begin{array}{*{20}c} {R = \frac{8\pi \mu L}{{A^{2} }} = \frac{8\mu L}{{\pi \left( {D/2} \right)^{4} }}} \\ \end{array} , $$

where $\mu$ is blood viscosity and $L$ is vessel length.

$D_{v}$ characterizes the degree of vasomotor activity. By substituting Eq. (5) into Eq. (2), $D_{v}$ can be expressed as:

6 $$ \begin{array}{*{20}c} {D_{v} = \sqrt {\frac{{\Delta {\mathrm{MAP}}_{r} *V_{{{\mathrm{meant}}}} }}{{\Delta {\mathrm{MAP}}_{t} *V_{{{\mathrm{meanr}}}} }}} } \\ \end{array} . $$

By further substituting Eq. (6) into Eq. (2), the resistance variation coefficient $R_{v}$ can be obtained:

7 $$ \begin{array}{*{20}c} {R_{v} = \left( {\frac{{\Delta {\mathrm{MAP}}_{t} *V_{{{\mathrm{meanr}}}} }}{{\Delta {\mathrm{MAP}}_{r} *V_{{{\mathrm{meant}}}} }}} \right)^{2} } \\ \end{array} . $$

C is used to evaluate the cerebrovascular capacity to regulate cerebral blood flow during the breath-hold task, reflecting the pressure–volume relationship and adaptability. It is calculated as [48]:

8 $$ \begin{array}{*{20}c} {C = \frac{\Delta V}{{\Delta {\mathrm{MAP}}^{\prime } }} = \frac{{\pi L\left( {\left( {D/2} \right)_{t}^{2} - \left( {D/2} \right)_{r}^{2} } \right)}}{{\Delta {\mathrm{MAP}}_{t} - \Delta {\mathrm{MAP}}_{r} }}} \\ \end{array} , $$

where $\Delta V$ is vascular volume change and is $\Delta MAP{\prime}$ pressure change.

### Experimental data analysis

A systematic analysis was conducted on cerebrovascular hemodynamic data collected bilaterally from 52 young volunteers, yielding a total of 104 datasets. The goal was to characterize heterogeneity in cerebrovascular regulation under hypercapnic conditions through multidimensional statistical approaches. All data processing and analyses were performed in MATLAB R2024a. The analytical workflow comprised statistical power assessment, distribution testing, correlation and group-difference analyses, and consistency validation. Prior to statistical analyses, all bilateral datasets (*n* = 104) underwent preprocessing and quality control. Abnormal values or recordings not meeting experimental criteria were excluded during acquisition to ensure that the final datasets reliably reflected physiological characteristics. According to the experimental design, the left and right cerebral arteries were analyzed as independent statistical units, thereby accounting for potential differences in vascular structure and regulatory responses.

#### Grouping criteria

Participants were grouped according to the magnitude of mean arterial pressure (MAP) changes induced by breath-holding. ΔBP was defined as the absolute change in MAP between post- and pre-breath-hold measurements, reflecting the intensity of the blood pressure response. Initially, quartile stratification [41–43] was employed, using the 25th percentile of ΔBP as a reference cutoff. Because ΔBP is a discrete variable and sample clustering occurred around 2 mmHg, the actual cumulative proportion at the 25th percentile slightly exceeded the theoretical 25%, which is a common feature in discrete distributions. Considering the measurement precision of the BP device (±0.5 mmHg) and the range of resting fluctuations, the final threshold was set at the 30th percentile corresponding to ΔBP = 2 mmHg to improve both physiological interpretability and statistical robustness. Based on this cutoff, participants were classified into the pressure-dependent group (ΔBP ≥ 2 mmHg) and the pressure-independent group (ΔBP < 2 mmHg). To further verify the robustness of the threshold selection, sensitivity analyses using the 25th and 35th percentiles of ΔBP were conducted, confirming consistency of results across different cutoff values.

To ensure sufficient statistical power for subgroup analyses, power analyses were performed using G*Power 3.1. For key hemodynamic parameters (*MAP*_*t*_ – *MAP*_*r*_, *R*_*v*_, and *D*_*v*_), independent two-sample t-tests (two-tailed, α = 0.05) were conducted, and effect sizes (Cohen’s d) along with corresponding statistical power (1–β) were calculated to confirm that the current sample sizes provided adequate power at the observed effect size levels.

#### Data preprocessing and quality control

Data preprocessing began with assessment of normality for all continuous variables. Normality was evaluated using the Kolmogorov–Smirnov test with Lilliefors correction, in combination with visual inspection of Q–Q plots to comprehensively examine distributional characteristics. Considering the sample size (*n* = 104), statistical tests may be sensitive to minor deviations from normality; therefore, both graphical patterns and the magnitude of deviation were taken into account in determining distributional assumptions. Overall, the principal hemodynamic variables demonstrated approximately normal distributions, satisfying the prerequisites for parametric statistical analyses.

For continuous variables showing approximate normality, Pearson correlation analysis was applied to quantify linear associations between indices. For variables with evident deviation from normality or not meeting parametric assumptions, Spearman rank correlation was employed to evaluate monotonic relationships.

To further verify measurement consistency and stability, Bland–Altman analyses were conducted for key parameters to assess limits of agreement and potential systematic bias. The vast majority of data points were located within the 95% limits of agreement, indicating that measurement variability remained within the acceptable range defined by the experimental design.

These preprocessing and quality control procedures ensured the scientific rigor and statistical robustness of the dataset, providing a reliable foundation for subsequent grouping analyses and investigations of hemodynamic associations.

#### Statistical analyses

For normally distributed variables, Pearson correlation was employed to assess the strength of linear relationships (r values; |r| < 0.1 = no correlation, 0.1–0.3 = weak, 0.3–0.5 = moderate, >0.5 = strong), with statistical significance determined at *p* < 0.01. For non-normally distributed variables, Spearman rank correlation coefficients (*ρ* values) were calculated to evaluate monotonic associations, while group differences were compared using the Mann–Whitney *U* test. Bland–Altman analyses were further applied to assess mean bias and 95% limits of agreement between paired measurements.

For parameters satisfying normal distribution, box plots were used to visualize data distributions, systematically identify skewness, outliers, and dispersion, and evaluate intergroup differences. Statistical significance was set at *α* = 0.05, with *p* < 0.05 considered significant. In addition, vascular compliance under different regulatory mechanisms was analyzed based on equation (8), providing critical insights into the mechanisms of vascular regulation.

In summary, the methodological framework integrated statistical inference with visual validation, offering a multilevel, scientifically rigorous, and reproducible approach to deciphering the diversity of cerebrovascular regulatory mechanisms.

## Data Availability

The datasets generated and/or analyzed during the current study are available from the corresponding author on reasonable request by qualified investigators.
